# CPR with restricted patient access using alternative rescuer positions: a randomised cross-over manikin study simulating the CPR scenario after avalanche burial

**DOI:** 10.1186/s13049-021-00944-9

**Published:** 2021-09-04

**Authors:** Bernd Wallner, Luca Moroder, Hannah Salchner, Peter Mair, Stefanie Wallner, Gabriel Putzer, Giacomo Strapazzon, Markus Falk, Hermann Brugger

**Affiliations:** 1 Department of Anaesthesiology and Intensive Care Medicine, Innsbruck Medical University Hospital, Medical University of Innsbruck, Anichstrasse 35, 6020 Innsbruck, Austria; 2 Institute of Mountain Emergency Medicine, Eurac Research, Viale Druso 1, 39100 Bolzano, Italy; 3 Department of Anaesthesiology and Critical Care Medicine, Hospital of Bolzano, Lorenz Böhler Strasse 5, 39100 Bolzano, Italy; 4 eScience, Sonnenstrasse 11, 39031 Bruneck, Italy

**Keywords:** Resuscitation, Confined space, Atypical rescuer position, Ventilation, Asphyxia

## Abstract

**Background:**

The aim of this manikin study was to evaluate the quality of cardiopulmonary resuscitation (CPR) with restricted patient access during simulated avalanche rescue using over-the-head and straddle position as compared to standard position.

**Methods:**

In this prospective, randomised cross-over study, 25 medical students (64% male, mean age 24) performed single-rescuer CPR with restricted patient access in over-the-head and straddle position using mouth-to-mouth ventilation or pocket mask ventilation. Chest compression depth, rate, hand position, recoil, compression/decompression ratio, hands-off times, tidal volume of ventilation and gastric insufflation were compared to CPR with unrestricted patient access in standard position.

**Results:**

Only 28% of all tidal volumes conformed to the guidelines (400–800 ml), 59% were below 400 ml and 13% were above 800 ml. There was no significant difference in ventilation parameters when comparing standard to atypical rescuer positions. Participants performed sufficient chest compressions depth in 98.1%, a minimum rate in 94.7%, correct compression recoil in 43.8% and correct hand position in 97.3% with no difference between standard and atypical rescuer positions. In 36.9% hands-off times were longer than 9 s.

**Conclusions:**

Efficacy of CPR from an atypical rescuer position with restricted patient access is comparable to CPR in standard rescuer position. Our data suggest to start basic life-support before complete extrication in order to reduce the duration of untreated cardiac arrest in avalanche rescue. Ventilation quality provided by lay rescuers may be a limiting factor in resuscitation situations where rescue ventilation is considered essential.

## Background

Current guidelines for companion rescue of completely buried avalanche victims recommend gaining access to the airway of the victim as quickly as possible. If snow obstructs the airway, it must be removed immediately and if there are no signs of life the victim should be completely extricated as quickly as possible and placed in the supine position for basic life support (BLS) [1]. However, recently published data show that completely extricating a victim for CPR in STA position takes five to twenty minutes [2]. Therefore, providing five rescue ventilations immediately after freeing the head and starting CPR before complete extrication in an atypical rescuer position may reduce duration of untreated cardiac arrest [3].

CPR may be challenging when performed in the confined space of a snow hole from an atypical rescuer position. So far, only little data is available on the feasibility and efficacy of chest compressions in an atypical rescuer position [4]. Previous work reported that the technique of over-the-head (OTH) CPR is able to provide external chest compression and manual ventilation close to the ERC Guidelines [5–7]. No study has so far evaluated the feasibility of OTH mouth-to-mouth-ventilation.

The aim of this manikin study was to evaluate the feasibility and efficacy of BLS performed in confined space using the OTH and straddle (STR) rescuer positions with both pocket mask and mouth-to-mouth ventilation.

## Material and methods

**Fig. 1:**
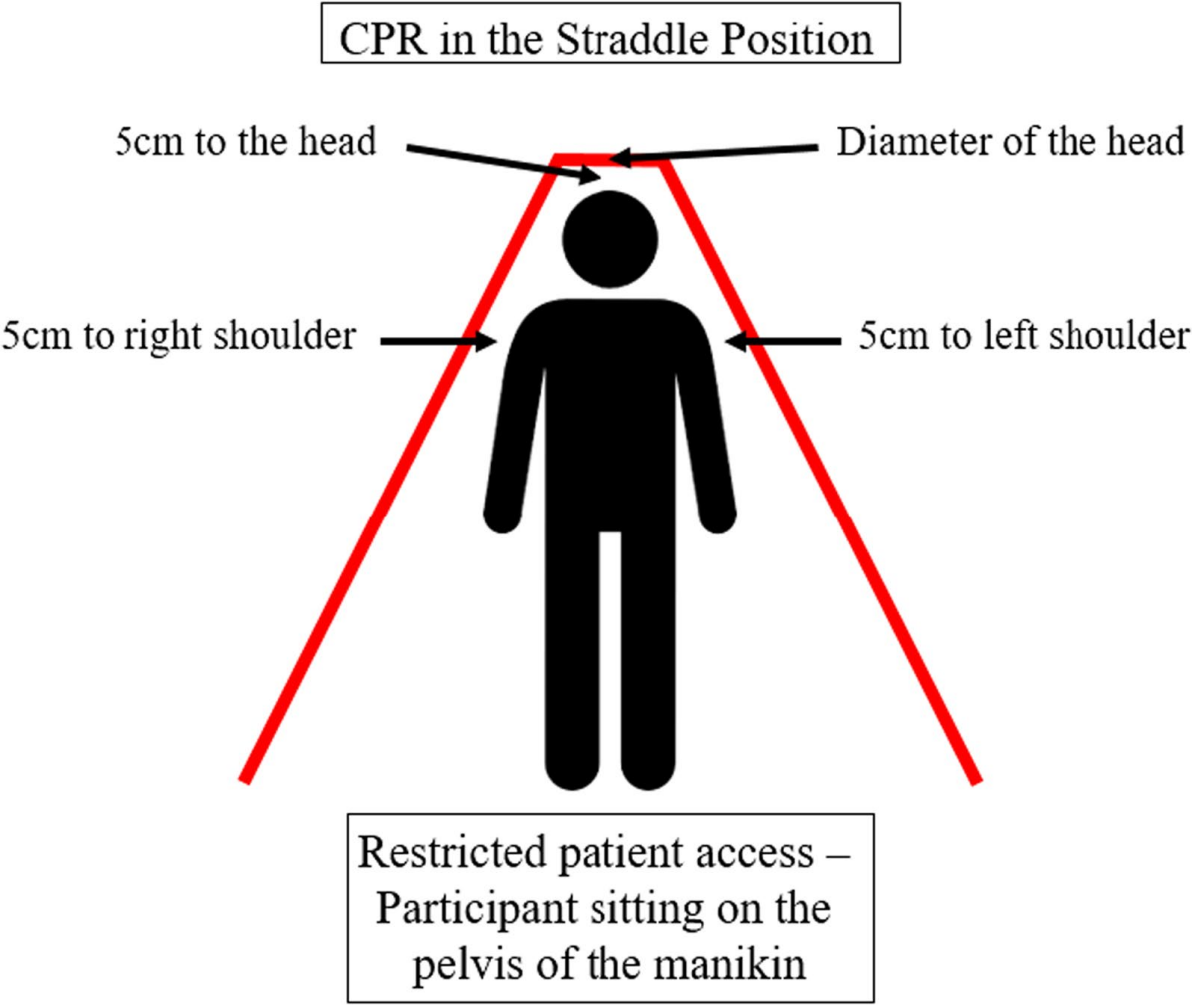
Setup for single-rescuer CPR in “straddle” position with restricted patient access. The participant sat on the pelvis of the manikin and remained in this position for the entire course of the experiment. Chest compressions and ventilation were provided by leaning forward towards the thorax and the mouth of the manikin

**Fig. 2:**
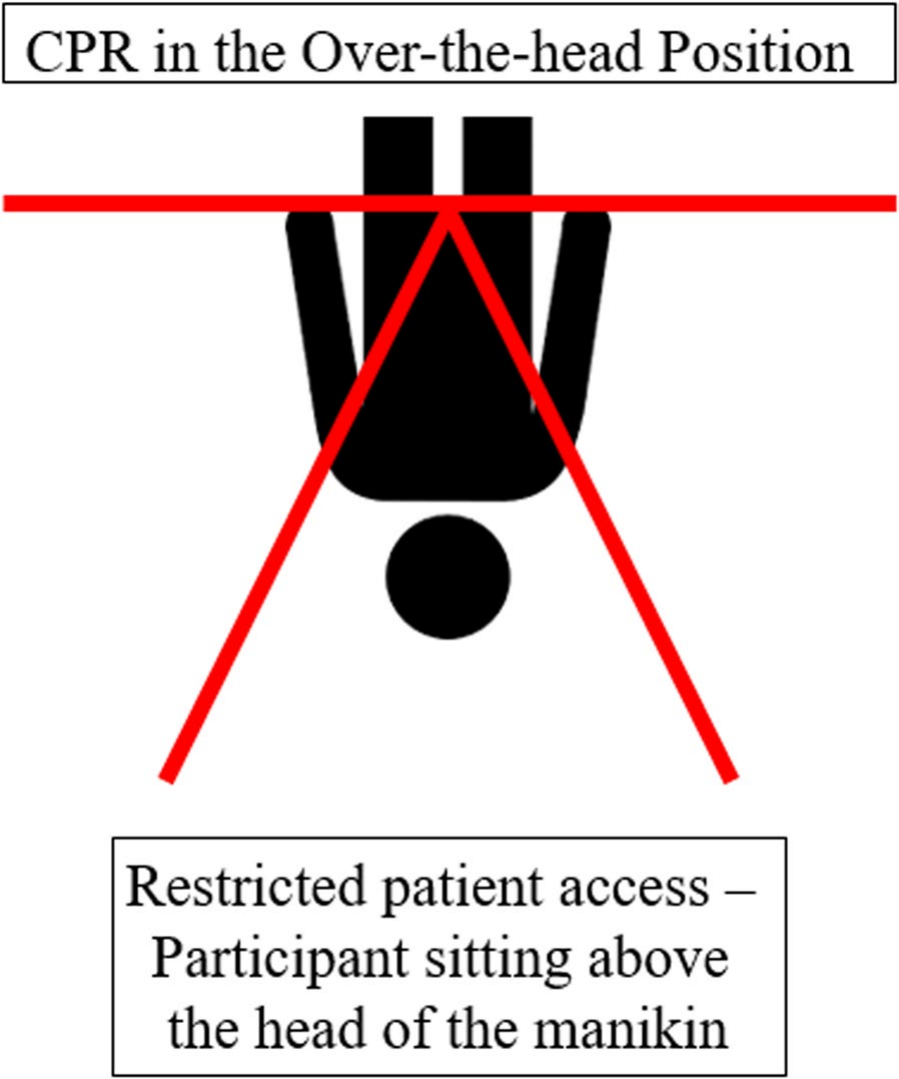
Setup for single-rescuer CPR in “over-the-head” position with restricted patient access. The participant kneeled above the manikin’s head and remained in this position for the entire course of the experiment. To perform chest compression the participant had to lean over the manikin’s head to reach the chest from above. In order to perform ventilation, the participant had to bend down to reach the mouth

This randomised, unblinded crossover manikin study compared single-rescuer BLS in standard position (STA) with single-rescuer CPR in STR and OTH position performed in a setting with confined space (Figs. 1, 2). Ventilation was performed using either face-shield mouth-to-mouth ventilation or pocket mask ventilation. The local ethics committee waived the need for approval.

### Participants and study site

For the study, 25 medical students from the Medical University of Innsbruck were recruited after completion of a 16-h BLS course, with most of them also completing a 16-h ALS-CPR course. Due to the sensitive electronics of the manikin, the study was not conducted in avalanche debris, but in a dry and temperature-controlled environment. Confined space was simulated with wooden panels in a standardized fashion (Figs. 1, 2). To limit available space for resuscitation, panels with a height of one meter were used, as this is the typical burial depth [8, 9] for avalanche victims (Figs. 1, 2).

### Study protocol

**Fig. 3:**
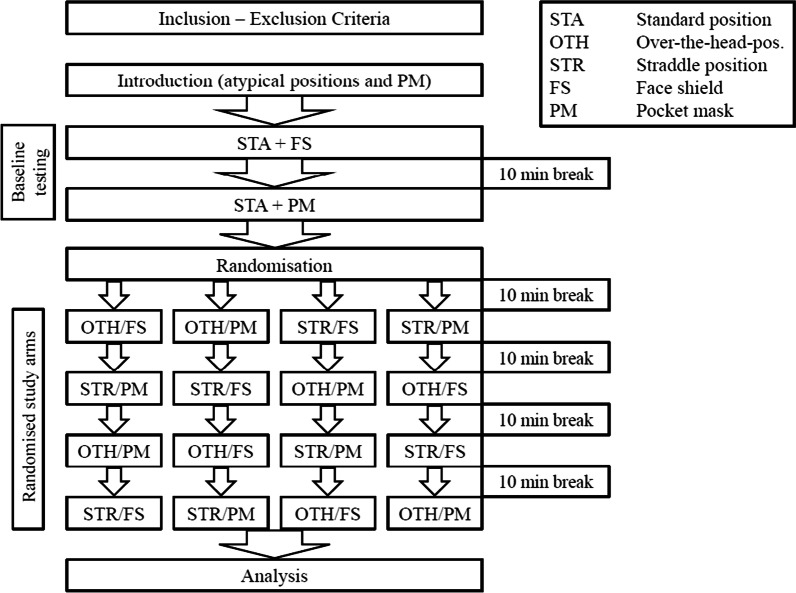
Flow chart displaying the details of the study protocol. After inclusion each participant received instructions, and subsequently performed two test cycles in the STA position (ventilation with FC and then BM). Thereafter, participants were randomised and performed BLS in the OTH and STR positions with FC or PM in random sequence

The study was performed as a prospective, randomised cross-over study. The sequence of the study protocol and flow diagram are shown in Fig. 3. Prior to trials, all participants completed a 20-min theoretical repetition lesson including guideline-compliant administration of initial rescue breaths, standard CPR cycles in a 30:2 compression to ventilation ratio, correct compression depth and compression rate. The two atypical CPR positions and the use of the pocket mask with the EC clamp technique were demonstrated to all participants by one of the authors. For the EC clamp technique one hand is placed so that the little, ring, and middle fingers are gripping the mandible from the angle to the chin and thereby forming the shape of the letter "E". The forefinger and thumb of the same hand form the letter “C” and press the mask onto the face to seal it tightly. However, prior to the trials there was no option to train pocket mask ventilation or CPR in atypical rescuer positions.

In a first step, baseline parameters were measured for each participant during a sequence of CPR (five rescue breaths followed by five cycles of CPR 30:2) in standard rescuer position. Baseline values were obtained for both mouth-to-mouth ventilation with a face shield (training shield, ref: 100,000,238, Austrian Red Cross) and ventilation with a pocket mask (Ambu ®, Res-Cue Mask TM, Ref: 000,252,206, Ambu A/S, Baltorpbakken 13, DK—2750 Ballerup, Denmark). The pocket mask was kept in place with the included rubber band, which the participant secured prior to the first ventilation. Thereafter, all participants performed CPR scenarios in atypical rescuer positions in a randomised sequence using mouth-to-mouth and pocket mask ventilation (Fig. 3). MTM was performed according to ERC guidelines [10, 11]. For MTM one hand was placed on the forehead and the other on the chin to achieve sufficient reclination. During PM ventilation a two-hands technique was used, thereby firmly holding the PM with two hands to fully cover mouth and nose and simultaneously recline the head of the manikin.

In “straddle” position the participant sat on the pelvis of the manikin, for the “over-the-head” position the participant kneeled behind the manikin’s head. Every participant performed a total of six trials. Between these trials, a minimum break of 10 min was allowed for physical recovery.

### Measurements

Chest compression depth, rate, hand position, recoil, chest compression/decompression ratio, hands-off times, tidal volume of ventilation and incidence of gastric insufflation were measured using a commercially available manikin (Ambu®ManWireless, A2344070000. Ambu A/S, Baltorpbakken 13, DK – 2750 Ballerup, Denmark).

The correct relationship between chest compression and decompression was defined as 40:60. Hands-off times were predefined by the manikin software as periods longer than nine seconds without chest compression.

### Statistics

Variables are given as mean and standard deviation. Statistical analysis was performed with Welch two-sample T-tests and Wilcoxon-Mann–Whitney tests (R version 3.6.0, Plating of a Tree, The R Foundation for Statistical Computing, University for Economics, Vienna, Austria). All given p-values are two-sided and values below 0.05 indicated statistical significance.

## Results

Mean age of the 25 participants was 24 years (SD + 2.4 years), 16 were male (64.0%), nine were female (36.0%). Twenty-three participants (92%) had completed both a BLS and an ALS-CPR course, two (8%) had completed only a BLS course. Of the participants 21 (84%) had no prior training with pocket mask ventilation.

### Chest compressions quality

Chest compression depth was greater than 50 mm in 98% of cases, regardless of rescuer position (Table 1). Mean chest compression rate was 124.7 compressions/minute ± 17. In 51 (34%) of the 150 CPR cycles, the compression rate was within the recommended ERC Guidelines range of 100–120 compressions per minute. Only eight (5.3%) of the 150 CPR cycles were performed with a rate of fewer than 100 compressions per minute (STA position 8.0%; OTH position 4.0%; STR position 4.0%). No difference in compression depth (*p* = 0.412) and rate (*p*  = 0.448) was seen between male and female participants (Wilcoxon-Mann–Whitney test). CPR cycles with an incorrect hand position were rare in all three rescuer positions (none in STA, 1 in OTH, 3 in STR position). Insufficient chest recoil was a common finding in 43.8% of all chest compressions with no significant difference between rescuer positions (42.7% in STA, 44.7% in OTH and 44.1% in STR position).

**Table 1:** Depth and rate of chest compressions during five consecutive CPR cycles in standard and atypical rescuer position

Rescuer position	Standard n = 7739*	Over-the-head n = 7791^a^	Straddle n = 7909^a^	Total n = 23,439^a^	Difference between groups
Compress. depth > 50 mm	n = 7663 (99. 0%)	n = 7721 (99.1%)	n = 7694 (96.14%)	n = 22,988 (98.08%)	*p* = 0.164
Compress. depth < 50 mm	n = 76 (1.0%)	n = 70 (0.9%)	n = 305 (3.86%)	n = 451 (1.92%)	*p* = 0.094
Insufficient chest recoil	n = 3306 (42.7%)	n = 3480 (44.7%)	n = 3489 (44.1%)	n = 10,275 (43.8%)	*p* = 0.322
Mean compression rate	122/min	128/min	124/min	125/min	*p* = 0.229

^a^The number of total chest compressions exceeded the number of chest compressions required by guidelines (total 22,500; 7500 in each position), because some participants performed more chest compressions than required

### Hands-off times

Overall hands-off times exceeding 9 s were found in 36.9% of 150 CPR cycles. Incidence of hands-off times did not significantly differ between the two ventilation techniques or the three rescuer positions studied (Table 3).

### Ventilation quality

Only 1400 (62.2%) of the 2250 ventilation attempts were detected by the manikin software. The number of guideline-conform (tidal volume between 400 and 800 ml), low-volume (tidal volume < 400 ml) and high-volume (tidal volume > 800 ml) ventilations is shown in Table 2; comparting the three rescuer positions and mouth-to-mouth to pocket mask ventilation. In 638 (45.6%) ventilations the tidal volume ranged between 400 and 800 ml, in 472 (33.7%) ventilations it was < 400 ml, and in 290 (20.7%) ventilations it was > 800 ml. (Table 2). In the remaining 850 (37.8%) ventilations the tidal volume was below 200 ml and the manikin thus did not record a sufficient attempt at ventilation. There was no significant difference in the portion of guideline-conform ventilations or mean tidal volume when comparing standard and atypical rescuer positions or mouth-to-mouth ventilation and pocket mask ventilation.

**Table 2:** Portion of insufficient, low-volume, guideline-conform and high-volume ventilations and mean tidal volume during initial rescue breaths and five consecutive CPR cycles

Rescuer position	Over-head position		Straddle position		Standard position		All positions	
Technique data available	MTM n = 375	PM n = 375	MTM n = 375	PM n = 375	MTM n = 375	PM n = 375	MTM^a^ n = 1,125	PM^a^ n = 1,125
Tidal volume 400–800 ml	n = 96 (25.6%)	n = 125 (33.3%)	n = 70 (18.7%)	n = 124 (33.1%)	n = 89 (23.7%)	n = 134 (35.7%)	n = 255 (22.7%)	n = 383 (34.0%)
Tidal volume < 400 ml	n = 89 (23.7%)	n = 76 (20.3%)	n = 58 (15.5%)	n = 83 (22.1%)	n = 66 (17.6%)	n = 100 (26.7%)	n = 213 (18.9%)	n = 259 (23.0%)
Tidal volume > 800 ml	n = 60 (16.0%)	n = 57 (15.2%)	n = 44 (11.7%)	n = 40 (10.7%)	n = 50 (13.3%)	n = 39 (10.4%)	n = 154 (13.7%)	n = 136 (12.1%)
No volume detected^a^	n = 130 (34.7%)	n = 117 (31.2%)	n = 203 (54.1%)	n = 128 (34.1%)	n = 170 (45.3%)	n = 102 (27.2%)	n = 503 (44.7%)	n = 347 (30.8%)
Tidal volume	400 ml ± 290 ml	450 ml ± 290 ml	390 ml ± 260 ml	480 ml ± 240 ml	400 ml ± 290 ml	450 ml ± 220 ml	400 ml ± 280 ml	500 ml ± 250 ml

Data for tidal volume given as mean and standard deviation of mean

MTM, mouth-to-mouth ventilation; PM, pocket mask ventilation

^a^Attempts at ventilation in which participants provided a tidal volume too small to be detected by the manikin

Of all ventilations (2250), 8.4% resulted in gastric insufflation. There was a non-significant trend toward reduced gastric insufflation overall using a pocket mask compared to mouth-to-mouth ventilation (2.2% vs 16.1%, *p* = 0.08). This lower rate of gastric insufflation was mainly due to significantly less gastric insufflation during pocket mask ventilation in the overhead position (*p* = 0.021) (Table 3).

**Table 3:** Incidence of gastric insufflation during 2250 attempts at ventilation (A) and incidence of hands-off times exceeding 9 s during 750 consecutive CPR cycles (B)

Position	Standard	Over-head	Straddle	All positions	*p* values
*Gastric insufflation*					
Mouth-to-mouth	n = 36 (17.6%)^a^	n = 47 (19.2%)^a^	n = 17 (9.9%)^a^	n = 100 (16.1%)^a^	*p* = 0.031
Pocket mask	n = 10 (3.7%)^a^	n = 1 (0.4%)^a^	n = 6 (2.5%)^a^	n = 17 (2.2%)^a^	*p* = 0.034
Both techniques	n = 46 (9.6%)^a^	n = 48 (9.5%)^a^	n = 23 (5.5%)^a^	n = 117 (8.4%)^a^	*p* = 0.045
*Hands-off time* > *9 s*					
Mouth-to-mouth	n = 46 (45.54%)	n = 45 (43.7%)	n = 45 (42.5%)	n = 136 (43.9%)	*p* = 0.904
Pocket mask	n = 29 (28.16%)	n = 29 (28.2%)	n = 34 (33.3%)	n = 92 (29.9%)	*p* = 0.646
All positions	n = 75 (36.8%)	n = 74 (35.9%)	n = 79 (38.0%)	n = 228 (36.9%)	*p* = 0.909

1400 ventilations were analysed (478 in standard position, 503 in OTH position and 419 in STR position). The number of ventilations available for analysis failed to reach the number of ventilations required by guidelines (2250), because some ventilations were too small to be detected

^a^% Percentage of gastric insufflation detected in the specific group

## Discussion

This randomized manikin trial suggests that OTH and STR positions during CPR in confined spaces allow the same effectiveness as CPR in the STA supine position. Our data reveal that ventilation is difficult to perform in all positions and may be a major limiting factor in the treatment of asphyctic cardiac arrest. The use of a pocket mask did not improve ventilation quality compared to mouth-to-mouth ventilation.

After avalanche burial, it may not be possible to start CPR in the STA position until the patient is fully extricated from the avalanche debris. However, to begin CPR as soon as possible, resuscitation efforts may need to be performed from alternative positions such as OTH or STR because of restricted patient access and non-supine position of an avalanche victim during rescue operations [2, 12]. Several studies investigating the feasibility of CPR in atypical rescuer positions demonstrated that high-quality chest compressions can be achieved despite challenging conditions [5, 7, 13–16], with one study even showing superior efficacy of chest compression in the OTH as compared to the STA position [7]. On the contrary, another study showed that BLS-trained flight attendants performed CPR in confined spaces more frequently with incorrect hand position and insufficient chest compressions in the OTH as compared to STA position [3]. In our study, we observed very high CPR efficiency in terms of chest compressions depth, rate and hand position regardless of the rescuer position suggesting that OTH and STR CPR have little effect on chest compression quality. We detected only minor deviations from guideline recommendations concerning compression rate and compression-decompression ratio, which were independent of rescuer position. However, we found a high overall rate of inadequate chest compression recoil. Possible reasons may be related to the confined space in our setting, where leaning may occur more easily and to the physical characteristics of our study participants. Insufficient chest recoil negatively impacts the outcome of cardiac arrest patients by reducing ventricular filling and cardiac output [17, 18], thus requiring greater attention in training and in real-life CPR scenarios.

Ventilation during BLS is difficult to perform for lay persons. Consequently, ventilation is losing priority in the BLS guidelines, where compression only CPR is favoured for sudden cardiac arrest [16]. Resuscitation guidelines recommend five initial rescue breaths in the case of asphyctic cardiac arrest [11, 19]. In the case of avalanche burial, the guidelines advise that access should be gained to the patient’s airway and airway and breathing immediately evaluated. Once the airway is freed from obstruction and apnoea is diagnosed the patient will benefit from immediate ventilation. In the case of asphyxia (e.g. in drowning) ventilation should be initiated immediately (e.g. while still in the water). The data from the current study provides evidence that immediately providing five rescue breaths in the case of an asphyxia-associated cardiac arrest is feasible, even before fully extricating the patient. After the five rescue breaths, further CPR including rescue breaths should be provided parallel to extrication from the avalanche.

Data on the effectiveness of rescue ventilation are conflicting, but most studies agree that sufficient ventilation is demanding, and efficacy varies markedly. This is even true for well-trained providers like professional medical personnel [7] and nurses [20]. Our results showing poor-quality ventilation are therefore not surprising, especially when considering that delivering efficient ventilation in complex rescue scenarios may be even more challenging. Conversely, the fact that neither OTH nor STR position further reduced ventilation quality was unexpected. For mouth-to-mouth ventilation one would expect major problems with ventilation in atypical rescuer positions and with restricted range of motion.

Previous studies investigated bag mask ventilation instead of mouth-to-mouth ventilation in OTH BLS scenarios, revealing superior efficacy of ventilation and a significantly larger proportion of correct ventilations in the OTH position [14, 19]. Available data indicate that using a mask and the EC clamp technique may facilitate superior ventilation in OTH position. The use of a mask and the EC clamp technique may explain the reduced rate of gastric insufflation observed in the current study in OTH position and pocket mask ventilation.

An increasing number of professional rescuers as well as lay persons are equipped with a pocket mask. Available data suggest that pocket mask ventilation may be superior to mouth-to-mouth ventilation, particularly in trained rescuers [20, 21]. Another study even recommend that single healthcare professional using a bag-valve-mask device should perform OTH CPR because of superior quality in CPR and ventilation [15].

Adelborg et al. investigated pocket mask and mouth-to-face-shield ventilation in lifeguards and found more guideline-conform ventilations, a higher tidal volume and shorter hands-off times when using a pocket mask [21]. Paal et al. also demonstrated higher tidal volumes with a pocket mask than with mouth-to-mouth ventilation with a concomitant lower incidence of gastric insufflation [20]. Theoretically, pocket mask ventilation offers additional advantages in alternative rescuer positions and situations when rescuer movement is restricted, although pocket mask ventilation has not been studied in such a scenario. We did not observe any improvement in tidal volume or rate of guideline-compliant ventilations when using a pocket mask, regardless of the rescuer position. However, we also demonstrated a lower incidence of gastric insufflation and shorter hands-off times than with mouth-to-mouth ventilation. Once secured to the face, the use of a pocket mask could facilitate neck hyperextension and may therefore improve ventilation during resuscitation in difficult conditions.

### Limitations

Due to the manikin susceptibility to moisture, investigation in an outdoor environment was not possible. Consequently, the restricted patient access encountered at the scene of avalanche extrication had to be simulated using wooden panels to make it as real as possible. Completely buried avalanche victims are found in prone position in 45% [12]. We could not investigate a scenario in prone position, since the manikin is certified only for supine position and the impossibility to ventilate patients discourage its application in avalanche-buried victims where asphyxia is the main cause of cardiac arrest. Aggravating environmental factors typically present at an avalanche burial site like wind and cold also could not be adequately simulated, nor could the extreme psychological stress, associated with avalanche burial. Furthermore, the significant physical burden associated with shovelling efforts during extrication before starting CPR was absent. It is possible that CPR performance in a real-life avalanche rescue situation is worse than observed in this study.

## Conclusion

Our results indicate that atypical rescuer positions during resuscitation in confined spaces do not worsen CPR quality as compared with resuscitation in the standard supine position. Thus, starting CPR before complete extrication may be a reasonable option for reducing duration of untreated cardiac arrest in avalanche rescue. Ventilation quality may be a limiting factor in bystander CPR scenarios after asphyxia-associated cardiac arrest. The use of a pocket mask did not improve ventilation quality in our study compared to mouth-to-mouth ventilation.

## Data Availability

The datasets used and analysed during the current study are available from the corresponding author (BW bernd.wallner@i-med.ac.at) on reasonable request.

## Abbreviations

CPR: Cardiopulmonary resuscitation
BLS: Basic life support
OTH: Over-the-head position
STR: Straddle position
STAL: Standard position
SD: Standard deviation
