# Correction to: CPR with restricted patient access using alternative rescuer positions: a randomised cross-over manikin study simulating the CPR scenario after avalanche burial

**DOI:** 10.1186/s13049-021-00957-4

**Published:** 2021-09-29

**Authors:** Bernd Wallner, Luca Moroder, Hannah Salchner, Peter Mair, Stefanie Wallner, Gabriel Putzer, Giacomo Strapazzon, Markus Falk, Hermann Brugger

**Affiliations:** 1 Department of Anaesthesiology and Intensive Care Medicine, Innsbruck Medical University Hospital, Medical University of Innsbruck, Anichstrasse 35, 6020 Innsbruck, Austria; 2 Institute of Mountain Emergency Medicine, Eurac Research, Viale Druso 1, 39100 Bolzano, Italy; 3 Department of Anaesthesiology and Critical Care Medicine, Hospital of Bolzano, Lorenz Böhler Strasse 5, 39100 Bolzano, Italy; 4 eScience, Sonnenstrasse 11, 39031 Bruneck, Italy

## Correction to: Scand J Trauma Resusc Emerg Med (2021) 29:129 https://doi.org/10.1186/s13049-021-00944-9

Following the publication of the original article [1], the authors requested to amend the “Acknowledgements” and “Funding” sections of their article, to acknowledge the financial support received by the Autonomous Province of Bozen/Bolzano.

The “Acknowledgements” section has now been updated to include the following statement: “The authors thank the Department of Innovation, Research, University and Museums of the Autonomous Province of Bozen/Bolzano for covering the Open Access publication costs”.

In the “Funding” section the sentence “There was no other funding supporting this manuscript” has been replaced by the sentence “The Department of Innovation, Research, University and Museums of the Autonomous Province of Bozen/Bolzano covered the Open Access publication costs”.

The original article has already been corrected as above.
